# Supplementary material for: Biallelic loss-of-function variants in DSCAM cause a neurodevelopmental syndrome with nystagmus and retinal dysfunction
Source: HGG Adv. 2026 Apr 30;7(3):100622. doi: 10.1016/j.xhgg.2026.100622 (PMC13199882; doi:10.1016/j.xhgg.2026.100622)
Supplement: Document S1. Figure S1 and Table S1 [file mmc1.pdf]

HGGA, Volume 7

Supplemental information

Biallelic loss-of-function variants in DSCAM cause a neurodevelopmental syndrome with nystagmus and retinal dysfunction

Sofia Douzgou Houge, Cecilie Bredrup, Ragnhild Wivestad Jansson, Ognjen Bojovic, Bayan M. Aljamal, Maha Al-Otaibi, Astrid S. Plomp, Mahdi M. Motazacker, Maria M. van Genderen, Anne Mellgren, Hisham Alkuraya, Omar Hikmat, Bjørn Ivar Haukanes, Fowzan S. Alkuraya, and Gunnar Douzgos Houge

Supplementary Figure S1: Location of the biallelic predicted loss-of-function variants in individuals 1 and 2 (dizygotic twins), individuals 4 and 5 (brothers), individual 3 (compound heterozygous for a frameshifting variant and an in-frame duplication of exons 2 and 3) and individual 6 (homozygous for a large deletion).

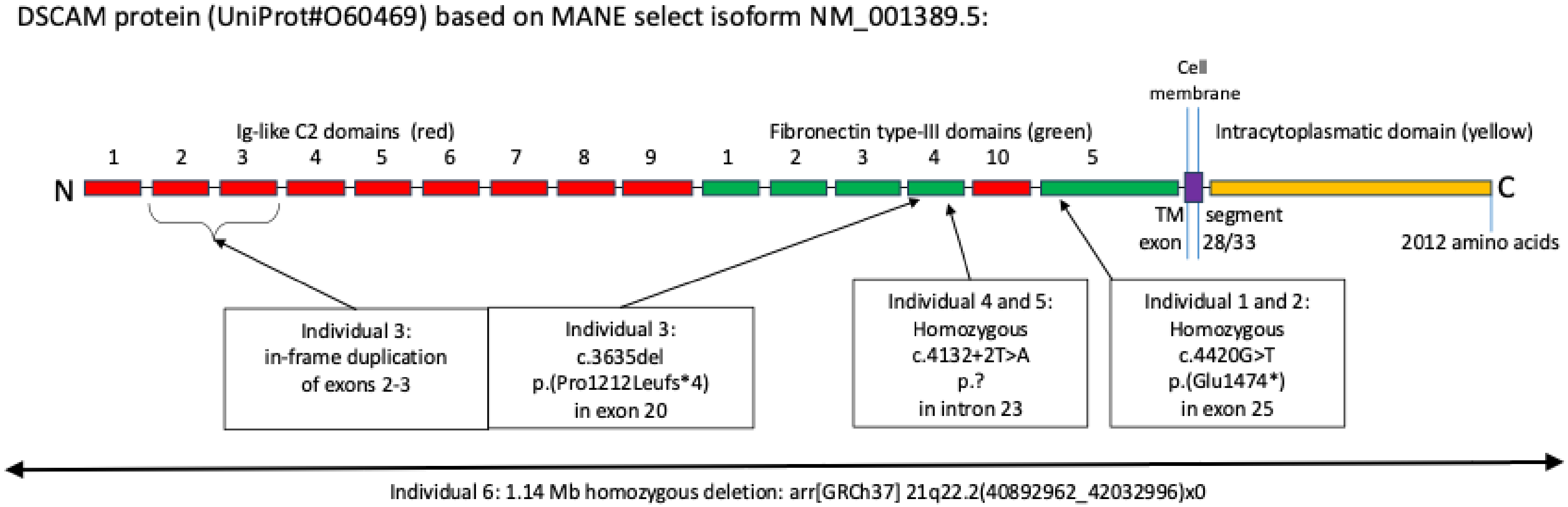

Supplementary Table S1: Neurodevelopmental testing data.

**Individual #**	**1**	**2**	**3**	
**Sex/age (years)**	F / 15 (DZ twin)	F / 15 (DZ twin)	M / 6	
**NM_001389.5 allele 1**	c.4420G>T	c.4420G>T	c.3635del	
**NM_001389.5 allele 2**	c.4420G>T	c.4420G>T	.arr[GRCh37] 21q22.2 (41969022_42168522)x3	
**Neurodevelopmental testing**	**Vineland – 3***	**Vineland – 3****	**Bayley-III scale at 31 months**	**Reynell-Zinkin scale at 31 months**
**Communication Domain**	25 (QI 20-30)	20 (QI 15-25)	Developmental age, 10 months<br>Language production, 10 months<br>Cognition, 10 months<br>Fine motor skills, 10 months<br>Language comprehension, 14 months	Language comprehension, 18-21 months<br>Language production, 9 months<br>Sensorimotor comprehension and exploration of surroundings, 15-18 months<br>Social adjustment, 12-15 months
**Daily Living Skills Domain**	41 (QI 37-45)	20 (QI 16-24)		
**Socialization Domain**	45 (QI 41-49)	37 (QI 33-41)		
**Adaptive Behavior Composite**	34 (QI 31-37)	24 (QI 21-27)		
**Other**	Paebody picture vocabulary test, 4th ed. 55 (<3SD)	Clinical Evaluation of Language Fundamentals, 4th ed., Score 1		

*Twin 1: Vineland- 3: all scores are under normal reference range and so full criteria for moderate intellectual disability

**Twin 2: Vineland- 3 scores: all scores are significantly under normal reference range and so full criteria for severe intellectual disability

Supplementary Electroretinogram (ERG) methodology

Photoreceptors convert light into electrical signals. Rods mediate scotopic (night) vision and outnumber cones by approximately 20:1, whereas cones mediate photopic vision (light-adapted visual acuity and color vision). The global electrical response of retinal neurons can be recorded by placing an electrode on the eye while stimulating the entire retina with standardized light flashes. This is the full field electroretinogram (ERG), and the preferred objective, non-invasive method for assessing overall outer retinal function.[1]

The initial negative deflection, the a-wave, reflects photoreceptor activity, followed by the positive b-wave, which primarily originates from the ON-bipolar cells. Waveform amplitude reflects the power of the responsive cells, whereas peak time better reflects their functional integrity. By testing under both dark and light adapted conditions, rod and cone pathways can be evaluated separately and the waveforms can be compared with normative age-matched data (displayed as rectangular boxes in Figure 3, with peak-time along the x-axis, and amplitude along the y-axis). Traditional ERG systems require a cooperative patient, but newer portable devices such as RETeval allow recordings in children and other difficult to test populations, with results comparable to standard systems.[2]

References:

1. Cornish EE, Vaze A, Jamieson RV, Grigg JR. The electroretinogram in the genomics era: outer retinal disorders. Eye (Lond). 35(9):2406-2418, 2021, PMID: 34234290

2. Carter P, Gordon-Reid A, Shawkat F, Self JE. Comparison of the handheld RETeval ERG system with a routine ERG system in healthy adults and in paediatric patients. Eye (Lond). 35(8):2180-2189, 2021, PMID: 33077909
