# Supplementary material for: Biallelic loss-of-function variants in DSCAM cause a neurodevelopmental syndrome with nystagmus and retinal dysfunction
Source: HGG Adv. 2026 Apr 30;7(3):100622. doi: 10.1016/j.xhgg.2026.100622 (PMC13199882; doi:10.1016/j.xhgg.2026.100622)
Supplement: Document S2. Article plus supplemental information [file mmc2.pdf]

HGG
Advances

REPORT

Biallelic loss-of-function variants in DSCAM cause a neurodevelopmental syndrome with nystagmus and retinal dysfunction

Sofia Douzgou Houge,[1,2,3] Cecilie Bredrup,[4,5] Ragnhild Wivestad Jansson,[4,5] Ognjen Bojovic,[1] Bayan M. Aljamal,[6] Maha Al-Otaibi,[7] Astrid S. Plomp,[8] Mahdi M. Motazacker,[8] Maria M. van Genderen,[9,10] Anne Mellgren,[4] Hisham Alkuraya,[11] Omar Hikmat,[12,13] Bjørn Ivar Haukanes,[1] Fowzan S. Alkuraya,[6,14,15] and Gunnar Douzgos Houge[1,13,16,17,*]

Summary

DSCAM occupies a 1-Mb locus in the original Down syndrome critical region on chromosome 21q22 and encodes a neuronal cell adhesion molecule of importance for brain and eye development. Singleton individuals, both born to first-cousin parents, with intellectual disability and homozygous *DSCAM* loss-of-function variants were reported in 2017 and in 2021, the latter also presenting with nystagmus and visual impairment. We present a cohort of five individuals, four new, including two sibling pairs with homozygosity or compound heterozygosity for predicted loss-of-function *DSCAM* variants. We identify a common clinical pattern of moderate to severe neurodevelopmental delay with poor language development, risk of focal seizures with onset in infancy, and nystagmus with poor vision. Electroretinography in two of the affected revealed cone-pathway dysfunction with a b-wave pattern indicating main dysfunction at the level of the cone-associated bipolar cells of the central retina. Our electroclinical findings are in line with previous *DSCAM* knockout chicken and mice studies that evidenced disturbed horizontal and vertical patterning of the retina. Taken together, we delineate a rare syndromic form of recessive intellectual disability with a distinctive type of visual impairment.

Down syndrome cell adhesion molecule (DSCAM [MIM: *602523]) is a neuronal adhesion molecule of the immunoglobulin superfamily with an unclear, if any, role in the pathogenesis of Down syndrome.[1,2] Other family members are the paralog genes *DSCAML1* (Down syndrome cell adhesion molecule like-1 [MIM:*611782]), *SDK1* (Sidekick cell adhesion molecule-1 [MIM:*607216]), and *SDK2* (Sidekick cell adhesion molecule-2 [MIM:*607217]). They all regulate horizontal and vertical patterning of the retina (i.e., the structuring of the retina into layers and columns) and are further important in the development of the rest of the nervous system.[3–7] Compound heterozygosity or homozygosity for likely loss-of-function (LoF) variants in *DSCAM* and *DSCAML1* is predicted to be a very rare event in outbred populations due to minor allele frequencies in the range of 1–2 per 10,000 (171 *DSCAM* LoFs and 273 *DSCAML1* LoFs are registered among the ~1,600,000 *DSCAM* alleles in gnomAD v4.1.0). Two individuals, both homozygous for predicted or definite LoF variants in *DSCAM*, have previously been reported: in 2017, Monies et al. published the results of exome-based sequencing of 1,000 Saudi Arabian families with a developmental disorder, which included an individual with neurodevelopmental delay (NDD), short stature, and seizures, homozygous for a canonical, likely pathogenic, splice-site variant in *DSCAM*(NM_001389.5):c.4132+2T>A (individual 16W-0265 in Table 4 of Monies et al.'s article).[8] Later, Hildebrandt et al. reported a 15-month-old girl homozygous for a 1.14-Mb 21q22.2 deletion removing *DSCAM* and three other genes (*B3GALT5* [MIM:*604066], *IGSF5* [MIM:*610638], and *PCP4* [MIM:*601629]) who had NDD, hypotonia, and nystagmus with poor vision.[9]

Here, we present four new individuals with predicted loss of DSCAM function due to nonsense, frameshift, or copy-number variants (Table 1). This study was performed according to the Declaration of Helsinki and approved by the Western Norway Regional Ethics Committee (REC 604007). Written informed consent for the publication of photographs and medical information was obtained from parents and/or legal guardians. We also present extended clinical information (individual 4, Table 1 and Figure 1D) regarding the homozygous individual published by Monies et al.[8] One of the new individuals is his younger brother, similarly affected with severe NDD, including poor language development and focal seizures,

[1]Department of Medical Genetics, Haukeland University Hospital, Bergen, Norway; [2]Frambu Unit, Norwegian Centre for Rare Diseases, Siggerud, Norway; [3]Brain Disorders Unit, Norwegian Centre for Rare Diseases, Oslo, Norway; [4]Department of Ophthalmology, Haukeland University Hospital, Bergen, Norway; [5]Department of Clinical Medicine (K1), University of Bergen, Bergen, Norway; [6]Department of Translational Genomics, Centre for Genomic Medicine, King Faisal Specialist Hospital and Research Centre, Riyadh, Saudi Arabia; [7]Department of Genetics, King Saud Medical City, Riyadh, Saudi Arabia; [8]Department of Human Genetics, Amsterdam University Medical Center, University of Amsterdam, Amsterdam, the Netherlands; [9]Bartiméus Diagnostic Center for Complex Visual Disorders, Zeist, the Netherlands; [10]Department of Ophthalmology, University Medical Center Utrecht, Utrecht, the Netherlands; [11]Global Eye Care, Specialized Medical Center Hospital, Riyadh, Saudi Arabia; [12]Department of Pediatric and Adolescent Medicine, Haukeland University Hospital, Bergen, Norway; [13]Department of Clinical Science (K2), University of Bergen, Bergen, Norway; [14]Lifera Omics, Riyadh, Saudi Arabia; [15]College of Medicine, Alfaisal University, Riyadh, Saudi Arabia; [16]Western Norway Precision Medicine Centre (NorPrem-HV), Haukeland University Hospital, Bergen, Norway
[17]Lead contact
*Correspondence: gunnar.douzgos.houge@helse-bergen.no
https://doi.org/10.1016/j.xhgg.2026.100622.
© 2026 The Author(s). Published by Elsevier Inc. on behalf of American Society of Human Genetics.
This is an open access article under the CC BY license (http://creativecommons.org/licenses/by/4.0/).

Table 1. Phenotypic features of biallelic DSCAM LoF

Individual #	1	2	3	4	5[8]	6[9]
Sex/age (years)	F/15 (DZ twin)	F/15 (DZ twin)	M/6	M/15	M/12 (sibling of #4)	F/1.5
**Genomic findings**[a]						
NM_001389.5 allele 1<br>NM_001389.5 allele 2	c.4420G>T<br>c.4420G>T (parents first cousins)	c.4420G>T<br>c.4420G>T (parents first cousins)	c.3635del<br>.arr[GRCh37] 21q22.2 (41969022_42168522)x3	c.4132 + 2T>A<br>c.4132 + 2T>A (parents first cousins)	c.4132 + 2T>A<br>c.4132 + 2T>A (parents first cousins)	arr[GRCh37] 21q22.2 (40892962_42032996)x0 (parents first cousins)
DSCAM allele 1<br>*DSCAM* allele 2	p.(Glu1474*)<br>p.(Glu1474*)	p.(Glu1474*)<br>p.(Glu1474*)	p.(Pro1212Leufs*4)<br>duplication of exons 2-3	p.?<br>p.?	p.?<br>p.?	deletion of *DSCAM*<br>deletion of *DSCAM*
**Clinical presentation**						
Pregnancy and birth	prolonged bleeding	ventriculomegaly	normal	normal	normal	na
Neurodevelopmental delay	Moderate	severe	moderate	moderate	moderate	yes
Language	2- or 3-word sentences	2- or 3-word sentences	nonverbal	2- or 3-word sentences	2- or 3-word sentences	yes
Hypotonia	Yes	yes	yes	N/A	N/A	yes
Seizures/EEG performed	focal/yes	no/yes	no/no	focal/no	focal/no	N/A
Age at sitting (years)	4	5	1.5	1.5	1	1.2
Age at walking (years)	6	8	4, with support	3	2	N/A
Regression of milestones	No	no	no	no	no	no
Behavior difficulties	No	no	no	aggression, ADHD	ADHD	N/A
Sleep disorder	difficulty falling asleep	no	no	N/A	N/A	N/A
Head circumference	normal	normal	normal	N/A	macrocephaly (+3.8 SD)	normal
Short stature (SD)	yes (−3.6)	yes (−3.9)	yes (−2)	yes (−2)	yes (−3.5)	normal (−1)
**Eyes**						
Visual acuity	poor (0.10)	poor (0.06)	poor (0.16)	poor	poor	poor
Strabismus	alternating esotropia	esotropia o.s.	esotropia o.d.	N/A	N/A	N/A
Refraction	+0.5 o.d./+2.5 o.s.	+1.0 o.u.	+4,5 o.u.	N/A	+3.0 o.u.	N/A
Photophobia	yes	yes	yes	N/A	N/A	N/A
Rotatory nystagmus	yes	yes	vertical/rotatory	yes	yes	yes
Fundus examination	apparently normal	parafoveal atrophy o.s.	scattered white dots in the mid periphery	N/A	scattered retinal lesions, normal macula	perifoveal atrophy o.s.
**Other findings**						
Brain MRI	small occipital lobe	ventriculomegaly	PWM anomalies	N/A	PWM anomalies	normal

(*Continued on next page*)

Table 1. *Continued*

Individual #	1	2	3	4	5[8]	6[9]
Pes planus	yes	yes	no	N/A	N/A	N/A
Toes	no	overlapping	short hallux	N/A	N/A	overlapping
Craniofacial dysmorphic features	yes	yes	yes	mild	mild	yes
Other genetic disease	Familial Mediterranean Fever (FMF, MIM #249100)	H syndrome (MIM: #602782)	no	N/A	N/A	N/A

ADHD, attention-deficit/hyperactivity disorder; N/A, no information available; PWM, periventricular white matter.
[a]Both whole-exome sequencing (WES) and genomic copy-number analysis were done in all individuals except individuals 4 and 5. In the latter, only WES was done.

and sharing the same *DSCAM* genotype (individual 5, Table 1 and Figure 1C). Both brothers also have rotatory nystagmus with poor vision. Fundus examination revealed a normal macula with scattered retinal lesions in the younger brother and was unsuccessful in the older one.

We also present 14-year-old dizygotic female twins, homozygous for a nonsense variant in *DSCAM*(NM_001389.5):c.4420G>T (p.Glu1474*) in exon 25 of 33 (individuals 1 and 2, Table 1; Figures 1A, 1B, and Figure S1). The twins were investigated by trio exome sequencing on the Illumina NextSeq 500 platform after exome selection by Roche Nimblegene SeqCap EZ MedExome kit. All exons of human *DSCAM* are highly conserved—unlike in *Drosophila*, where extensive alternative splicing is the mode of functional regulation.[10] Since exon 25 is in frame, exon skipping is theoretically possible but functionally also likely to be detrimental.

The twins have moderate and severe NDD with severely delayed language development and short stature (Tables 1 and S1). Neurological examination of individual 1 revealed generalized hypotonia. Deep tendon reflexes were present but difficult to elicit. Assessment of coordination was abnormal, with impaired performance on finger-to-nose testing, heel-to-shin maneuver, and rapid alternating movements (dysdiadochokinesia). She had bilateral pes planus and ambulated with an upright posture and a broad-based gait, balance impairment, and difficult-to-achieve tandem gait. She experienced focal evolving to bilateral tonic clonic seizures with onset at age 1 year. An electroencephalogram (EEG) performed at 9 years of age revealed findings consistent with focal seizures localized to the posterior region of the left hemisphere. The seizures were well controlled with lamotrigine (twice daily). Individual 2 had seizures during early childhood that resolved (normal EEG results by the age of 15 years) and similarly abnormal gait.

Although visual impairment may have contributed to difficulty in gait and coordination seen in individual 1, a few findings support a primary neurological contribution. The presence of generalized hypotonia, reduced reflex excitability, and abnormal coordination across multiple tests indicated dysfunction beyond visual impairment, as these tasks assess motor planning, proprioception, and cerebellar integration rather than visual input exclusively. Furthermore, impaired performance on toe and heel walking and the presence of a broad-based gait with balance instability suggested an underlying central motor component. In addition, bilateral pes planus represented a structural and postural abnormality unlikely to be secondary to visual dysfunction.

Both twins manifested photophobia, mixed rotatory nystagmus, and an upward-gaze tendency from early infancy. There has been no evidence of deterioration of visual function, and they continue to utilize vision for orientation. Individual 1 has no problems in the dark and presents with esotropia and moderate hyperopia

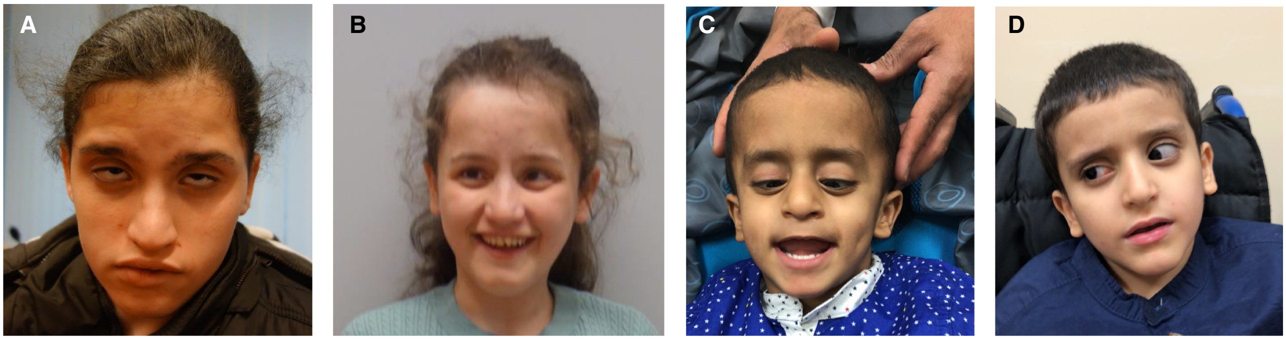

Figure 1. Facial photographs
Facial photographs of the dizygotic twins, individuals 1 (A) and 2 (B), and the brothers, individuals 5 (C) and 4 (D). Lack of eye contact due to rotatory nystagmus. Mildly dysmorphic craniofacial features: prominent glabella, narrow nasal root, bulbous nasal tip, and square chin.

(+3 −1 180 both eyes). Individual 2 is suspected to have left-sided amblyopia secondary to anisometropia and left esotropia (+1.5 right eye, +4 −2.5 20 left eye). Composite visual function is 0.10 in twin 1 and 0.06 in twin 2 (Snellen). The siblings show a consistent preference for holding near-vision devices, such as tablets and smartphones, at a very close distance. Despite the reduced distance vision, they can recognize close relatives at approximately 4–5 m. Pupillary reflexes are normal, and the ocular media are clear with unremarkable corneas, lenses, and anterior chambers. Ophthalmoscopy has been unsuccessful due to involuntary eye movements, but widefield fundus photography (poor quality) of the right eye in individual 1 showed no gross structural abnormalities. Individual 2, who has the poorest vision, was examined under general anesthesia to assess retinal structure and function. Ophthalmoscopy and retinal imaging with RetCam showed normal optic disks and retinal vessels; no evident diabetic retinopathy; but some small, discrete grayish retinal lesions in the lower midperiphery, unremarkable macula right eye, and parafoveal atrophy of the left macula (Figure 2) resembling the retinal phenotype described in individual 6 (Table 1).

Full-field electroretinography (ERG) (RETeval with skin electrodes, see supplementary methodology) in individuals 2 and 3 revealed relatively preserved rod-pathway function but marked cone-pathway dysfunction, consistent with the clinical presentation of intact night vision, but pronounced photophobia, nystagmus, and reduced visual acuity (Figure 3). In individual 2, the scotopic dim flash response, dark adapted (DA) 0.01 (reflecting rod-pathway function) was in the lower normal range, with borderline delayed b-wave (Figure 3 row A). However, the a- and b-waves of the scotopic bright-flash response, DA 3.0, originating mainly from rod photoreceptors and rod bipolar cells, respectively, were within normal limits, indicating preserved rod-pathway function (Figure 3 row B). In contrast, the photopic (light-adapted) ERG, reflecting cone-pathway function, revealed a low and borderline delayed a-wave (cone photoreceptor response) with bright-light stimulation, light-adapted (LA) 3.0, and a profoundly delayed and attenuated b-wave both with bright-light (Figure 3 row C) and flicker stimulation, 28.3-Hz flicker (Figure 3 row D), suggesting main dysfunction at the level of the cone-associated bipolar cells.

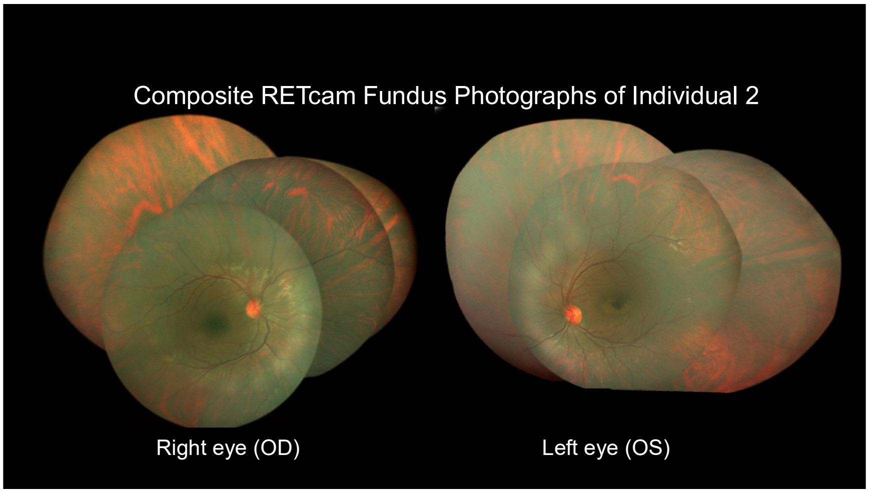

Figure 2. Composite fundus photographs of the right and left retina of patient 2 obtained under general anesthesia using a RetCam fundus camera
Both eyes show normal optic disks, retinal vessels, and peripheral retina with choroidal translucency and no evidence of diabetic retinopathy. Small grayish retinal lesions in the lower midperiphery and nasal to the optic disks. The left eye demonstrates a parafoveal macular atrophy, whereas the right macula appears normal.

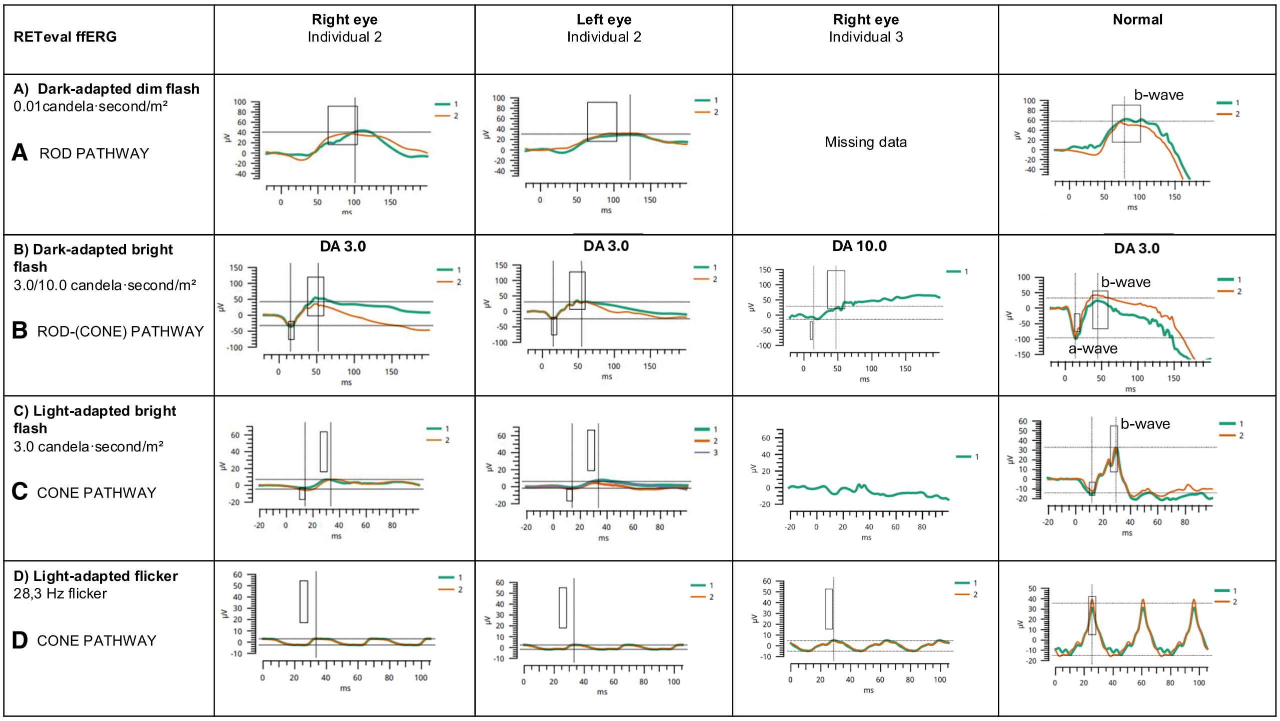

Figure 3. Electrophysiological recordings from both eyes of patient 2 (under general anesthesia) and the right eye of patient 3 (limited protocol without sedation) obtained using the RETeval hand-held ERG system with skin electrodes, compared with normal reference traces

(A and B) Dark-adapted, scotopic responses (DA 0.01, DA 3.0, and DA10.0) were largely within the normal range, indicating substantial preservation of rod-pathway function.

(C and D) Light-adapted photopic responses (LA 3.0- and LA 28.3-Hz flicker) show marked impairment of the cone pathway, particularly evident in the attenuated b-wave amplitude and delayed peak times in the LA 3.0- and LA 28.3-Hz flicker traces.

Of note, each twin also has one additional recessive genetic finding inherited from both parents that is unlikely to contribute to the loss-of-*DSCAM*-related phenotype. Individual 1 is homozygous for a known pathogenic missense variant in *MEFV*(NM_000243.2):c.2230G>T (p.Ala744Ser) and has no symptoms of familiar mediterranean fever (FMF; [MIM: 249100]) to date. Individual 2 is homozygous for a pathogenic frameshift variant in *SLC29A3*(NM_001363518.1):c.54del (p.Ser19Glnfs*4), causing histiocytosis-lymphadenopathy plus syndrome (H syndrome [MIM: 602782]). This manifested, at 12 years of age, with an intradural spinal tumor of mature B and T lymphocytes that was surgically removed, diabetes mellitus type 1, and pancreatic insufficiency.

Finally, we report the findings of a 6-year-old boy, compound heterozygous for a frameshift variant in exon 20 and an intragenic in-frame duplication of exons 2 and 3 (of 33): *DSCAM*(NM_001389.5):c.3635del (p.Pro1212Leufs*4) and .arr[GRCh37]21q22.2(41969022_42168522)x3 (individual 3, Table 1 and Figure S1). We have not verified that this is a classical tandem duplication that adds 165 amino acids (amino acid 15–170) to the conserved extracellular immunoglobin-like domain of DSCAM, but, if this is the case, an LoF effect is likely. This individual was nonverbal until age 4.5 years; at 6 years he has single, intelligible words and communicates using a speech computer. Neurodevelopmental tests evidence moderate NDD/intellectual disability (Table S1). He walked with support at age 4 years. He has mild short stature with normal head circumference. He also has mixed vertical/rotatory nystagmus with poor visual attention (also suggesting cerebral visual impairment). Fundus examination was normal except for a few scattered white dots in the mid periphery. The visual acuity has been stable: at age 2 and 3 years it was 20/260 and at age 4 and 6 years it was 20/125 (measured with Teller Acuity Cards with symbols that children can recognize). The visual field was measured with confrontational methods and showed intact peripheral vision when he had visual attention. At age 6 years, a limited ERG was performed (individual 3, Figure 3). The DA bright-flash signal, DA10.0, was within the normal range and did not show an electronegative waveform (Figure 3 row B). For the LA test (or cone-pathway test), results were outside the normal range with a strongly reduced LA 3.0 cd.s/m^2 response (Figure 3 row C) and a delayed and attenuated 28.3-Hz flicker response with an abnormal wave shape (Figure 3 row D). Overall, the results were in line with the ones obtained in individual 2.

All six individuals, homozygous or compound heterozygous inherited predicted or definite LoF variants (ACMG/AMP class PVS1) in *DSCAM*, have moderate-to severe NDD

with poor language development. Reported onset of ambulation varied from 2 to 8 years, and in 4/6 hypotonia was reported. Short stature was a feature in 5/6, including the individual born to non-consanguineous parents. Focal seizures was a feature in 3/6. The most remarkable common feature is rotatory/vertical nystagmus with poor vision, making retinal structural and functional assessment difficult in addition to the moderate to severe NDD. In both individuals with ERG findings, the rod pathway seemed functional while the cone pathway was dysfunctional, predominantly at the level of the cone-associated bipolar cells of the retina (Figure 3). This affection of cone-related bipolar cells fits well with human and mouse single-cell expression data.[11,12] *DSCAM* is expressed in retinal ganglion cells, amacrine cells, and cone-related bipolar cells, with the strongest expression in the latter (see web resources for details).

Our electroclinical findings fit well with data from chicken that showed that *DSCAM* is essential for proper organization of the inner plexiform layer of the retina that connects the bipolar cells to the retinal ganglion cells,[3] and knockout (KO)-mice studies that showed that *DSCAM* is needed for self-neuronal avoidance in the retina, necessary to ensure proper neurite arborization and mosaic spacing of similar types of retinal neurons.[4,13,14] Lack of such neuronal self-avoidance not only disrupts the evenly spaced horizontal mosaic pattern of retinal neurons but also makes these neurons clump together and avoid apoptosis despite self-aggregation.[13] The latter could relate to the transcriptional effect of the intracellular domain of DSCAM that can be cleaved off by γ-secretase and affect transcription of genes associated with apoptosis, neuronal differentiation, and synapse function.[15] Lack of DSCAM can thus promote retinal ganglion cell survival. Data from conditional Dscam KO mice suggests that DSCAM has an important role in retinal organization only at an early stage of development.[13] Congenital rotatory nystagmus, photophobia, and reduced visual acuity with no apparent progression during childhood and adolescence in two of the oldest patients in this cohort support a developmental origin of the retinal dysfunction detected by ERG, mainly affecting the cone pathway. Constitutional loss of DSCAM had minimal effect on forebrain development in KO mice, including lamination, patterning and connectivity, but they did have motor coordination defects and seizures,[5] also described in this cohort.

DSCAM has low biological tolerance for both LoF and missense variation (gnomAD v4.1.0: *DSCAM* pLI 1.00 [o/e 0.22], *Z* score 4.45). An association with *de novo* variants in *DSCAM* has been observed in two trio exome sequencing studies of large cohorts of simplex families each having a child with an autism spectrum disorder (ASD).[16,17] Induced pluripotent stem (iPS)-derived neuronal cells from an individual with ASD indicated that less DSCAM was associated with downregulation of NMDA receptors in the synapse with diminished NMDA-receptor-mediated currents, and data from heterozygous KO mice supported this association.[6] The heterozygote parents of all newly reported individuals here do not have any neurodevelopmental or ocular phenotype and are clinically normal. Monoallelic mutations in *DSCAM* may be potential contributors to neurodevelopmental disorders; however, further studies including in-depth clinical characterization are necessary for conclusions regarding this.

In summary, we provide clinical evidence for a recessive syndromic condition characterized by moderate to severe neurodevelopmental delay with poor language development, risk of focal seizures with onset in infancy, hypotonia, short stature, mildly dysmorphic craniofacial features, gait abnormalities, poor vision, and rotatory/vertical nystagmus with retinal dysfunction.

Data and code availability

The published article includes all data generated during this study. The ClinVar accession numbers for the unpublished variants in Table 1 are SCV007541355–SCV007541358.

Acknowledgments

We are most grateful to the participating families. We thank optometrist Mauricio Arturo Suncin Aguilar for his assistance. This work has been generated within Project 2629394, The post-exome clinic: improving the impact of exome sequencing for developmental disorders in Norway, funded by the Norwegian Centre for Rare Diagnoses, and the Undiagnosed Project 358387, funded by the Research Council of Norway (grants to S.D.H. and G.D.H.) and within the European Reference Network on Rare Congenital Malformations and Rare Intellectual Disability (ERN-ITHACA) (EU Framework Partnership Agreement ID: 3HP-HP-FPA ERN-01-2016/739516). Funders played no role in study design, data collection, analysis and interpretation of data, or the writing of this manuscript.

Author contributions

Conceptualization, S.D.H. and G.D.H.; data curation, C.B., O.B., B.I.H., B.M.A., A.S.P., and R.W.J.; formal analysis, C.B., R.W.J., B.I.H., and G.D.H.; funding acquisition, S.D.H. and G.D.H.; investigation, C.B., O.B., B.M.A., M.A.-O., A.M., H.A., R.W.J., B.M.A., A.S.P., M.M.M., M.M.v.G., O.H., F.S.A., and S.D.H.; methodology, S.D.H. and G.D.H.; project administration, S.D.H. and G.D.H.; supervision, S.D.H., C.B., and F.S.A.; visualization, C.B., O.B., R.W.J., M.M.v.G., S.D.H., and G.D.H.; writing – original draft, S.D.H., C.B., and G.D.H.; writing – review and editing, all authors.

Declaration of interests

The authors declare no competing interests.

Web resources

Genome Aggregation Database, https://gnomad.broadinstitute.org/

Online Mendelian Inheritance in Man, http://www.omim.org/

Single-cell atlas of the human retina from CZI cell science, https://cellxgene.cziscience.com/collections/4c6eaf5c-6d57-4c76-b1e9-60df8c655f1e

Single-cell atlas of the mouse retina from Broad Institute, https://singlecell.broadinstitute.org/single_cell/study/SCP2560/mrca-scrna-seq-of-the-mouse-retina-all-cells

Supplemental information

Supplemental information can be found online at https://doi.org/10.1016/j.xhgg.2026.100622.

Received: January 17, 2026
Accepted: April 24, 2026

References

1. Weisfeld-Adams, J.D., Tkachuk, A.K., Maclean, K.N., Meeks, N.L., and Scott, S.A. (2016). A de novo 2.78-Mb duplication on chromosome 21q22.11 implicates candidate genes in the partial trisomy 21 phenotype. npj Genom. Med. *1*, 16003. https://doi.org/10.1038/npjgenmed.2016.3.
2. Ronan, A., Fagan, K., Christie, L., Conroy, J., Nowak, N.J., and Turner, G. (2009). Familial 4.3 Mb duplication of 21q22 sheds new light on the Down syndrome critical region. BMJ Case Rep. *2009*, bcr05.2009.1914. https://doi.org/10.1136/bcr.05.2009.1914.
3. Yamagata, M., and Sanes, J.R. (2008). Dscam and Sidekick proteins direct lamina-specific synaptic connections in vertebrate retina. Nature *451*, 465–469. https://doi.org/10.1038/nature06469.
4. Fuerst, P.G., Koizumi, A., Masland, R.H., and Burgess, R.W. (2008). Neurite arborization and mosaic spacing in the mouse retina require DSCAM. Nature *451*, 470–474. https://doi.org/10.1038/nature06514.
5. Mitsogiannis, M.D., Pancho, A., Aerts, T., Sachse, S.M., Vanlaer, R., Noterdaeme, L., Schmucker, D., and Seuntjens, E. (2020). Subtle Roles of Down Syndrome Cell Adhesion Molecules in Embryonic Forebrain Development and Neuronal Migration. Front. Cell Dev. Biol. *8*, 624181. https://doi.org/10.3389/fcell.2020.624181.
6. Lim, C.S., Kim, M.J., Choi, J.E., Islam, M.A., Lee, Y.K., Xiong, Y., Shim, K.W., Yang, J.E., Lee, R.U., Lee, J., et al. (2021). Dysfunction of NMDA receptors in neuronal models of an autism spectrum disorder patient with a DSCAM mutation and in Dscam-knockout mice. Mol. Psychiatry *26*, 7538–7549. https://doi.org/10.1038/s41380-021-01216-9.
7. Fuerst, P.G., Harris, B.S., Johnson, K.R., and Burgess, R.W. (2010). A novel null allele of mouse DSCAM survives to adulthood on an inbred C3H background with reduced phenotypic variability. Genesis *48*, 578–584. https://doi.org/10.1002/dvg.20662.
8. Monies, D., Abouelhoda, M., AlSayed, M., Alhassnan, Z., Alotaibi, M., Kayyali, H., Al-Owain, M., Shah, A., Rahbeeni, Z., Al-Muhaizea, M.A., et al. (2017). The landscape of genetic diseases in Saudi Arabia based on the first 1000 diagnostic panels and exomes. Hum. Genet. *136*, 921–939. https://doi.org/10.1007/s00439-017-1821-8.
9. Hildebrandt, C., Fulton, A., and Rodan, L.H. (2021). Homozygous deletion of 21q22.2 in a patient with hypotonia, developmental delay, cortical visual impairment, and retinopathy. Am. J. Med. Genet. *185*, 555–560. https://doi.org/10.1002/ajmg.a.61969.
10. Guo, L., Wu, Y., Chang, H., Zhang, Z., Tang, H., Yu, Y., Xin, L., Liu, Y., and He, Y. (2021). Structure of cell-cell adhesion mediated by the Down syndrome cell adhesion molecule. Proc. Natl. Acad. Sci. USA *118*, e2022442118. https://doi.org/10.1073/pnas.2022442118.
11. Li, J., Wang, J., Ibarra, I.L., Cheng, X., Luecken, M.D., Lu, J., Monavarfeshani, A., Yan, W., Zheng, Y., Zuo, Z., et al. (2026). Single-cell atlas of the the trancriptome and chromatine accessibility in the human retina. Nat Genet *58*, 418–433. https://doi.org/10.1038/s41588-025-02454-1.
12. Li, J., Choi, J., Cheng, X., Ma, J., Pema, S., Sanes, J.R., Mardon, G., Frankfort, B.J., Tran, N.M., Li, Y., and Chen, R. (2024). Comprehensive single-cell atlas of the mouse retina. iScience *27*, 109916. https://doi.org/10.1016/j.isci.2024.109916.
13. Fuerst, P.G., Bruce, F., Rounds, R.P., Erskine, L., and Burgess, R.W. (2012). Cell autonomy of DSCAM function in retinal development. Dev. Biol. *361*, 326–337. https://doi.org/10.1016/j.ydbio.2011.10.028.
14. Huberman, A.D. (2009). Mammalian DSCAMs: they won't help you find a partner, but they'll guarantee you some personal space. Neuron *64*, 441–443. https://doi.org/10.1016/j.neuron.2009.11.011.
15. Sachse, S.M., Lievens, S., Ribeiro, L.F., Dascenco, D., Masschaele, D., Horré, K., Misbaer, A., Vanderroost, N., De Smet, A.S., Salta, E., et al. (2019). Nuclear import of the DSCAM-cytoplasmic domain drives signaling capable of inhibiting synapse formation. EMBO J. *38*, e99669. https://doi.org/10.15252/embj.201899669.
16. Wang, T., Guo, H., Xiong, B., Stessman, H.A.F., Wu, H., Coe, B.P., Turner, T.N., Liu, Y., Zhao, W., Hoekzema, K., et al. (2016). De novo genic mutations among a Chinese autism spectrum disorder cohort. Nat. Commun. *7*, 13316. https://doi.org/10.1038/ncomms13316.
17. Iossifov, I., O'Roak, B.J., Sanders, S.J., Ronemus, M., Krumm, N., Levy, D., Stessman, H.A., Witherspoon, K.T., Vives, L., Patterson, K.E., et al. (2014). The contribution of de novo coding mutations to autism spectrum disorder. Nature *515*, 216–221. https://doi.org/10.1038/nature13908.

HGGA, Volume 7

Supplemental information

Biallelic loss-of-function variants in DSCAM cause a neurodevelopmental syndrome with nystagmus and retinal dysfunction

Sofia Douzgou Houge, Cecilie Bredrup, Ragnhild Wivestad Jansson, Ognjen Bojovic, Bayan M. Aljamal, Maha Al-Otaibi, Astrid S. Plomp, Mahdi M. Motazacker, Maria M. van Genderen, Anne Mellgren, Hisham Alkuraya, Omar Hikmat, Bjørn Ivar Haukanes, Fowzan S. Alkuraya, and Gunnar Douzgos Houge

Supplementary Figure S1: Location of the biallelic predicted loss-of-function variants in individuals 1 and 2 (dizygotic twins), individuals 4 and 5 (brothers), individual 3 (compound heterozygous for a frameshifting variant and an in-frame duplication of exons 2 and 3) and individual 6 (homozygous for a large deletion).

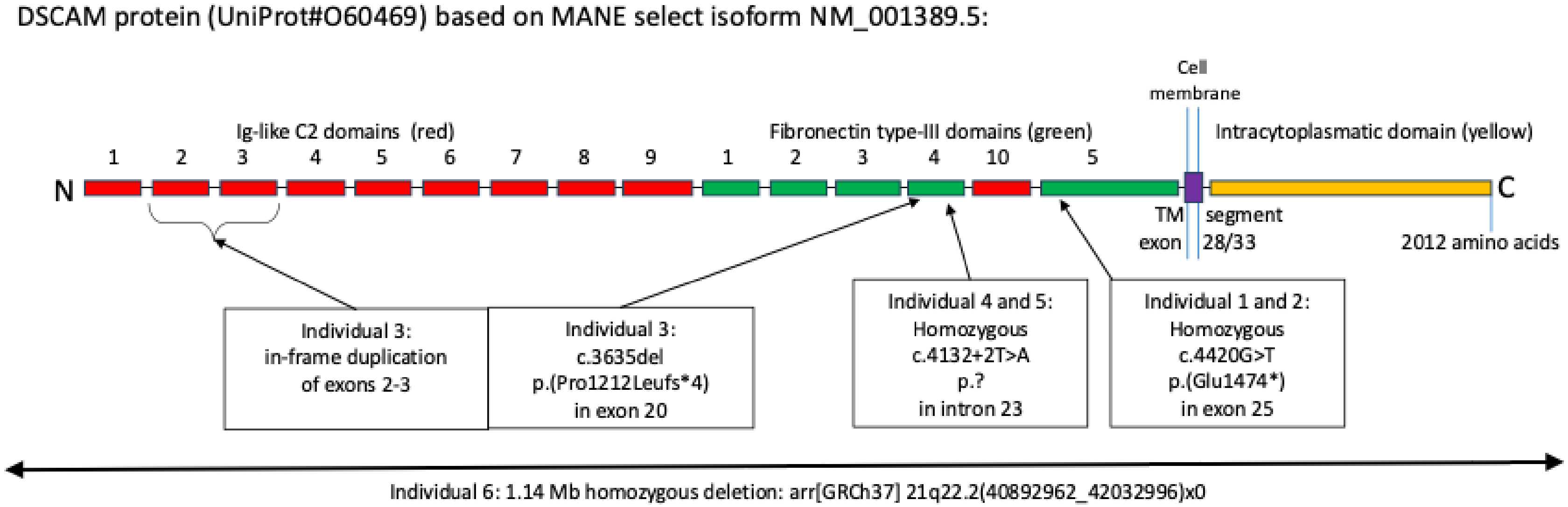

Supplementary Table S1: Neurodevelopmental testing data.

Individual #	1	2	3	
**Sex/age (years)**	F / 15 (DZ twin)	F / 15 (DZ twin)	M / 6	
**NM_001389.5 allele 1**	c.4420G>T	c.4420G>T	c.3635del	
**NM_001389.5 allele 2**	c.4420G>T	c.4420G>T	.arr[GRCh37] 21q22.2 (41969022_42168522)x3	
**Neurodevelopmental testing**	**Vineland – 3***	**Vineland – 3****	**Bayley-III scale at 31 months**	**Reynell-Zinkin scale at 31 months**
**Communication Domain**	25 (QI 20-30)	20 (QI 15-25)	Developmental age, 10 months<br>Language production, 10 months<br>Cognition, 10 months<br>Fine motor skills, 10 months<br>Language comprehension, 14 months	Language comprehension, 18-21 months<br>Language production, 9 months<br>Sensorimotor comprehension and exploration of surroundings, 15-18 months<br>Social adjustment, 12-15 months
**Daily Living Skills Domain**	41 (QI 37-45)	20 (QI 16-24)		
**Socialization Domain**	45 (QI 41-49)	37 (QI 33-41)		
**Adaptive Behavior Composite**	34 (QI 31-37)	24 (QI 21-27)		
**Other**	Paebody picture vocabulary test, 4th ed. 55 (<3SD)	Clinical Evaluation of Language Fundamentals, 4th ed., Score 1		

*Twin 1: Vineland- 3: all scores are under normal reference range and so full criteria for moderate intellectual disability

**Twin 2: Vineland- 3 scores: all scores are significantly under normal reference range and so full criteria for severe intellectual disability

Supplementary Electroretinogram (ERG) methodology

Photoreceptors convert light into electrical signals. Rods mediate scotopic (night) vision and outnumber cones by approximately 20:1, whereas cones mediate photopic vision (light-adapted visual acuity and color vision). The global electrical response of retinal neurons can be recorded by placing an electrode on the eye while stimulating the entire retina with standardized light flashes. This is the full field electroretinogram (ERG), and the preferred objective, non-invasive method for assessing overall outer retinal function.[1]

The initial negative deflection, the a-wave, reflects photoreceptor activity, followed by the positive b-wave, which primarily originates from the ON-bipolar cells. Waveform amplitude reflects the power of the responsive cells, whereas peak time better reflects their functional integrity. By testing under both dark and light adapted conditions, rod and cone pathways can be evaluated separately and the waveforms can be compared with normative age-matched data (displayed as rectangular boxes in Figure 3, with peak-time along the x-axis, and amplitude along the y-axis). Traditional ERG systems require a cooperative patient, but newer portable devices such as RETeval allow recordings in children and other difficult to test populations, with results comparable to standard systems.[2]

References:

1. Cornish EE, Vaze A, Jamieson RV, Grigg JR. The electroretinogram in the genomics era: outer retinal disorders. Eye (Lond). 35(9):2406-2418, 2021, PMID: 34234290

2. Carter P, Gordon-Reid A, Shawkat F, Self JE. Comparison of the handheld RETeval ERG system with a routine ERG system in healthy adults and in paediatric patients. Eye (Lond). 35(8):2180-2189, 2021, PMID: 33077909
